# CD33‐targeting extracellular vesicles deliver antisense oligonucleotides against FLT3‐ITD and miR‐125b for specific treatment of acute myeloid leukaemia

**DOI:** 10.1111/cpr.13255

**Published:** 2022-07-18

**Authors:** Huan Chen, Migara Kavishka Jayasinghe, Eric Yew Meng Yeo, Zhiyuan Wu, Marco Pirisinu, Waqas Muhammad Usman, Thach Tuan Pham, Kah Wai Lim, Nhan Van Tran, Anskar Y. H. Leung, Xin Du, Qiaoxia Zhang, Anh Tuân Phan, Minh T. N. Le

**Affiliations:** ^1^ Department of Pharmacology, Institute for Digital Medicine, Yong Loo Lin School of Medicine National University of Singapore Singapore Singapore; ^2^ Department of Surgery, Immunology Program, Cancer Program and Nanomedicine Translational Program, Yong Loo Lin School of Medicine National University of Singapore Singapore Singapore; ^3^ Department of Biomedical Sciences, Jockey Club College of Veterinary Medicine and Life Sciences City University of Hong Kong Kowloon Hong Kong SAR; ^4^ Division of Physics & Applied Physics, School of Physical & Mathematical Sciences Nanyang Technological University Singapore Singapore; ^5^ Department of Medicine, Li Ka Shing Faculty of Medicine, Queen Mary Hospital The University of Hong Kong Pok Fu Lam Hong Kong SAR; ^6^ Department of Hematology and Shenzhen Bone Marrow Transplantation Public Service Platform, Shenzhen Second People's Hospital The First Affiliated Hospital of Shenzhen University, Shenzhen University School of Medicine Shenzhen China; ^7^ Present address: Jotbody Ltd, Science Park New Territories Hong Kong SAR

## Abstract

**Introduction:**

Acute Myeloid Leukaemia (AML) is the most common blood cancer in adults. Although 2 out of 3 AML patients go into total remission after chemotherapies and targeted therapies, the disease recurs in 60%–65% of younger adult patients within 3 years after diagnosis with a dramatically decreased survival rate. Therapeutic oligonucleotides are promising treatments under development for AML as they can be designed to silence oncogenes with high specificity and flexibility. However, there are not many well validated approaches for safely and efficiently delivering oligonucleotide drugs. This issue could be resolved by utilizing a new generation of delivery vehicles such as extracellular vesicles (EVs).

**Methods:**

In this study, we harness red blood cell‐derived EVs (RBCEVs) and engineer them via exogenous drug loading and surface functionalization to develop an efficient drug delivery system for AML. Particularly, EVs are designed to target CD33, a common surface marker with elevated expression in AML cells via the conjugation of a CD33‐binding monoclonal antibody onto the EV surface.

**Results:**

The conjugation of RBCEVs with the CD33‐binding antibody significantly increases the uptake of RBCEVs by CD33‐positive AML cells, but not by CD33‐negative cells. We also load CD33‐targeting RBCEVs with antisense oligonucleotides (ASOs) targeting *FLT3‐ITD* or miR‐125b, 2 common oncogenes in AML, and demonstrate that the engineered EVs improve leukaemia suppression in in vitro and in vivo models of AML.

**Conclusion:**

Targeted RBCEVs represent an innovative, efficient, and versatile delivery platform for therapeutic ASOs and can expedite the clinical translation of oligonucleotide drugs for AML treatments by overcoming current obstacles in oligonucleotide delivery.

## Graphical abstract

In this study, we harness red blood cell‐derived EVs (RBCEVs) and engineer them with surface functionalization and exogenous drug loading to develop an efficient drug delivery system for AML. Anti‐CD33 antibody was conjugated to RBCEVs using an enzymatic method combined with the streptavidin‐biotin system. We load the antibody conjugated RBCEVs with ASOs targeting FLT3‐ITD or miR‐125b, 2 common oncogenes in AML, and demonstrate that the treatment with engineered EVs improve leukaemia suppression both in vitro and in vivo.

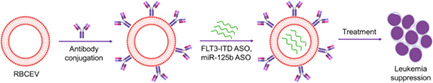

## 1 INTRODUCTION

AML is one of the most common haematopoietic malignancies, according to the American Cancer Society, with estimated 20,050 new cases and 11,540 deaths in the United States in 2022.^1^ Although many therapies are available for treating AML, their ability to halt disease progression remains limited due to multiple mechanisms of drug resistance that leukaemia cells develop with time.^2^ Chemotherapeutic agents such as cytarabine and daunorubicin remain the standard of care for early‐stage AML with a complete remission rate of 60%–80%.^3^ Alternatively, patients with adverse prognostic features will undergo allogeneic stem cell transplantation, depending on cytogenetic factors and the availability of donors. Though these treatment regimens are based on the outcome of early clinical trials, they lack the consideration of disease heterogeneity and often lead to the emergence of therapy‐resistant leukaemic clones with an overall cure rate of 30%–40%.^3^, ^4^ A large number of patients with resistance to standard chemotherapy die of progressive disease or chemotherapy‐induced toxicity of repeated treatments.^4^ The recent advances in sequencing methods unwind the heterogeneity of AML and reveal several intrinsic and acquired mutations which eventually lead to relapse and treatment failure.^4^ Hence, there is an urgent need to develop novel and effective targeted therapeutic strategies to overcome these resistance mechanisms and improve treatment outcomes for AML. RNA drugs including siRNAs, miRNAs, and ASOs are validated approaches for silencing gene expression to treat diseases with different clinical outcomes.^5^, ^6^ RNA drugs are an innovative tool that can be designed to modulate any gene of interest, therefore, effective even against targets otherwise unreachable with conventional small therapeutic molecules.^7^ The versatility of this method offers an essential aid to treat diseases with evolving mutations such as cancer or viral‐infectious diseases.^8^ However, RNA drugs are susceptible to degradation, clearance, and the induction of immune responses.^9^, ^10^ Hence, they require chemical modifications or carriers to prevent their degradation and ensure efficient delivery into target cells.

Nano‐sized particles, such as liposomes, have been engineered as nanocarriers to enhance the targeted delivery of therapeutic molecules.^11^, ^12^ However, synthetic nanoparticles are limited by various problems such as toxicity and immunogenicity.^13^, ^14^ Extracellular vesicles (EVs) are natural nano‐sized vesicles containing bioactive molecules which are capable of inducing phenotypic changes in recipient cells upon uptake.^15^ EVs display surface proteins and antigens from parental cells that confer stealth properties, stability, and increased half‐life.^16^ The discovery of EVs as natural RNA carriers is a fundamental breakthrough in nanomedicine.^17^ Indeed, the possibility of repurposing natural mechanisms of intercellular communication into new therapeutic strategies is an approach that may revolutionize future medical approaches.

However, endogenous unmodified EVs show poor therapeutic potential and low target specificity. In this regard, EVs have been surface engineered to display targeting moieties on their surface, conferring improved target specificity, and biodistribution. In this respect, genetic engineering of donor cells is routinely used to generate EVs expressing proteins able to recognize specific target cells.^18^ However, if stably transduced cell lines are employed to produce EVs, abundant oncogenic factors including mutant DNA, RNA, and proteins may be packaged in EVs and delivered to recipient cells, leading to an increased risk of tumorigenesis.

Our research group recently standardized and validated a platform to purify large‐scale quantities of red blood cell‐derived EVs (RBCEVs) (10^13^–10^14^ EVs from each blood unit) from human red blood cells in a low cost and time‐efficient fashion.^19^ We have shown in our previous study that a variety of RNA therapeutics including ASOs, gRNAs, and Cas9 mRNA could be loaded into RBCEVs and subsequently delivered to target cells, showing significant therapeutic effects in vitro and in in vivo models of breast cancer and AML.^19^ Moreover, as RBCs lack nuclei, which precludes the use of genetic engineering methods using plasmids, we have developed a novel strategy for stable and biocompatible conjugation of EVs with targeting moieties. This method employs protein ligases that create covalent bonds between specially designed peptides and proteins on the surface of EVs, enabling stable conjugation without any damage to EVs. Using a linker peptide, we have also demonstrated the ability to conjugate EVs with proteins such as single‐domain antibodies. We have shown that EVs conjugated with a peptide or a single domain antibody targeting EGFR promote the specific uptake of EVs by EGFR‐positive lung cancer cells, thereby enhancing the delivery of paclitaxel to lung tumours for better treatment of lung cancer.^20^

In this study, we sought to develop targeted delivery of ASOs using RBCEVs for AML treatment. We improved the engineering approach of RBCEVs by conjugating the RBCEV surface with an anti‐CD33 antibody. CD33‐targeting RBCEVs specifically accumulated in CD33‐positive AML cells and enabled CD33‐specific delivery of RNA cargo. We observed the enhanced efficacy of CD33‐targeting RBCEVs loaded with ASOs in suppressing oncogenic FLT3‐ITD or miR‐125b levels in AML cells in vitro, resulting in a decreased cell viability. Furthermore, we demonstrated an enhanced leukaemia suppression of an AML cell line xenograft model using FLT3‐ITD ASO loaded CD33‐targeting RBCEVs and in patient‐derived xenograft models of AML using miR‐125b ASO loaded CD33‐targeting RBCEVs. Hence, this study presents a new advancement in EV‐based therapeutics for targeted treatment of AML.

## 2 MATERIALS AND METHODS

### 2.1 Cell culture

Acute myeloid leukaemia MOLM13 cells were obtained from DSMZ Collection of Microorganisms and Cell Cultures (Braunschweig, Germany). HEK‐293T cells and acute lymphoblastic leukaemia CEM cells were obtained from the American Type Culture Collection (ATCC, USA). Luciferase‐GFP‐labelled MOLM13 (MOLM13‐Luc‐GFP) cells were generated by infecting MOLM13 cells with pCAG‐Fluc‐eGFP lentivirus and sorted using flow cytometry. Generation of MOLM13‐Luc‐GFP and CEM cell lines were maintained in RPMI1640 (ThermoFisher Scientific, USA) supplemented with 10% heat‐inactivated fetal bovine serum (Biosera, USA) and 1% penicillin/streptomycin (ThermoFisher Scientific, USA) and cultured at 37°C with 5% CO_2_ in a tissue culture incubator.

### 2.2 Purification of RBCEVs

Blood samples were provided byhealthy donors with informed consent. Briefly, 200–250 ml of group O blood was collected in single system blood bags (Macopharma, France). Blood samples were processed according to approved guidelines after all related procedures were approved. RBCs were separated from plasma by centrifugation (1000 ×*g* for 8 min at 4°C) followed by three‐times washing with PBS at the same speed. The few white blood cells existing in RBCs were removed by leukodepletion filter (Nigale, China). The purified RBCs were placed in Nigale buffer and diluted up to three times in PBS containing 0.1 mg/ml calcium chloride and 10 μM calcium ionophore (Sigma, USA) and incubated overnight at 37°C with 5% CO_2_ to induce vesiculation. RBCEVs were purified according to our previous study.^19^ Purified RBCEVs were stored in PBS supplemented with 4% trehalose at −80°C.

### 2.3 Characterization of RBCEVs

For transmission electron microscopy analysis, RBCEVs were washed with size exclusion chromatography (SEC) and diluted to a concentration of 1.0 mg/ml. The RBCEVs were diluted 1:1 with 4% paraformaldehyde (PFA) for 10 min at room temperature (final PFA concentration is 2%). Following fixation, the RBCEVs were immobilized onto a glow discharged copper grid coated with formvar carbon film (Electron Microscope Science, USA) by incubating for 5 min. Excess RBCEVs were blotted off and the grids were rinsed twice with PBS. This was followed up with a final rinse in distilled water. RBCEVs on the grid were incubated with 3% uranyl acetate for 5 min to perform negative staining of RBCEVs. This was followed by a brief rinse with distilled water to remove excess uranyl acetate stain to obtain better contrast between background and EVs. The grids were air dried for 10 min before being imaged using a Tecnai G2 transmission electron microscope (FEI, USA) at 100 kV.

Nanoparticle tracking analysis of EVs was performed using a Zetaviewer nanoparticle tracking analysis system (Particle Metrix, Germany). Briefly, EVs were diluted 10,000‐fold in filtered PBS. The resultant EVs were injected into a pre‐calibrated Zetaviewer and analysed across 11 frames for size and concentration. Filtered PBS was used as a blank control.

### 2.4 Enzyme expression and purification

BL21 (DE3) *E. coli* bacteria were transformed with OaAEP1‐Cys247Ala plasmid (provided by Dr. Bin Wu, Nanyang Technology University) followed by a Kanamycin selection. Lysogeny broth (LB) medium containing 0.4 mM IPTG was used to induce protein expression at 16°C, 250 RPM for 18 h. The bacteria were collected by centrifugation at 8000× *g* for 15 min at 4°C and resuspended in 50 ml binding buffer (500 mM NaCl, 25 mM Tris–HCl, 5% glycerol, 1 mM phenylmethylsulfonyl fluoride sulfonyl fluoride). The bacteria suspension was passed through a high‐pressure homogenizer at 1000 psi for 4–6 cycles for lysis. The protein supernatant was collected by centrifugation at 10,000× *g* for 60 min at 4°C followed by filtration through 0.45 μm syringe filters (Millipore, USA). The inactive OaAEP1‐ Cys247Ala was purified from the supernatant by an NGC‐QUEST‐10 fast protein liquid chromatography (FPLC) system (Bio‐Rad, USA) combined with a 5‐ml‐Ni‐charged cartridge (Bio‐Rad), using the detailed procedure as described in our previous study.^20^ For OaAEP1 ligase activation, the inactive enzyme was incubated in 200 mM acetate buffer supplemented with 1 mM EDTA and 0.5 mM Tris (2‐carboxyethyl) phosphine hydrochloride. The mixture was adjusted to a pH of 3.7 and incubated for 10 days at 4°C to cleave the inhibitory cap domain of the enzyme. The activated proteins were dialyzed with a 3‐kDa‐cutoff concentrator and stored at a concentration of 20 μM at −80°C.

### 2.5 Conjugation of EVs with antibodies

The anti‐human CD33 and IgG isotype control monoclonal antibodies were conjugated onto RBCEVs surface through enzymatic ligation of biotinylated peptide and the streptavidin‐biotin approach. The first step was OaAEP1‐mediated ligation of biotinylated TR5 peptide (B‐TL5) with RBCEVs. 50 μg RBCEVs were incubated with 500 μM B‐TL5, and 2 μM OaAEP1 ligase in PBS buffer (pH 6.5) at room temperature for 3 h. RBCEVs were washed with PBS using qEV SEC columns and 3 times centrifugation at 21,000× *g* for 20 min. Next, biotinylated peptide ligated RBCEVs were incubated with 0.1 mg/ml streptavidin (ThermoFisher) at room temperature for 2 h, followed by extensive washing to remove excess streptavidin. For the conjugation of RBCEVs with antibodies, biotinylated anti‐human CD33 (BioLegend, USA Cat# 366628) or mouse IgG1 κ isotype control monoclonal antibodies (BioLegend, USA Cat#400103) were incubated with streptavidin‐conjugated RBCEVs at a concentration of 0.1 mg/ml at room temperature for 2 h, to conjugate biotinylated antibodies onto RBCEVs through the streptavidin‐biotin system. RBCEVs were washed 3 times via centrifugation at 21,000× *g* for 20 min to remove unbound antibodies.

### 2.6 ASO design and synthesis

For FLT3‐ITD ASO, a series of 16‐nucleotide (nt) ASOs were designed to specifically target the repeated region of *FLT3‐ITD*. The ASOs have a 3‐10‐3 gapmer configuration (i.e. 3‐nt locked nucleic acid [LNA]‐modified ends flanking a central 10‐nt DNA segment) and are fully modified with phosphorothioate (PS) chemistry throughout the backbone. All FLT3‐ITD ASOs were synthesized in‐house with an ABI 394 DNA/RNA synthesizer on Glen UnySupport (Glen Research) using standard phosphoramidite chemistry. LNA phosphoramidites purchased from Sigma‐Aldrich, and phenylacetyl disulfide (ChemGenes Corporation) was used as the sulfurizing reagent. Oligonucleotide cleavage and deprotection were performed under concentrated aqueous ammonia at 55°C for 16 h. The ASOs were purified with Poly‐Pak II cartridges (Glen Research) following the manufacturer's protocol, desalted using Glen Pak 2.5 desalting column (Glen Research), and dried by lyophilization. The ASOs were characterized by JEOL SpiralTOF matrix‐assisted laser desorption/ionization time‐of‐flight (MALDI‐TOF) mass spectrometer.

miR‐125b ASO and FAM‐conjugated ASO (FAM ASO) were synthesized by Shanghai Genepharma (Shanghai, China) as described in our previous paper.^19^

### 2.7 ASO loading and quantification

ASOs were loaded into RBCEVs using REG1 (Carmine Therapeutics, Singapore) according to the manufacturer's protocol. ASO‐loaded RBCEVs were washed 3 times with PBS using centrifugation at 21,000× *g* for 20 min. To quantify the ASO loading efficiency, 50 μg ASO‐loaded RBCEVs were suspended in 7 μl nuclease‐free water followed by adding 2 μl 1% Triton X‐100 buffer to lyse RBCEVs. 6× DNA loading dye (New England Biolabs, USA) was added into the mixture before loading and running the samples on 2% agarose gel. A serial dilution of free ASO from 1 μg to 0.0625 μg was used to plot a standard curve for determining the quantity of ASO loaded into RBCEVs. Bio‐Rad ChemiDoc gel documentation system was used for imaging.

### 2.8 In vitro uptake and delivery of RBCEVs

To test the uptake specificity of RBCEVs, MOLM13 and CEM cells were seeded at 5 × 10^4^ cells per well in 48‐well plates 8 h in advance and treated with 50 μg carboxyfluorescein diacetate succinimidyl ester (CFSE, ThermoFisher)‐labelled CD33‐targeting/non‐targeting RBCEVs for 2 h at 37°C. The cells were subsequently collected and washed twice with cold FACS buffer followed by flow cytometry analysis.

To test the targeted delivery of ASOs by RBCEVs, FAM ASO was loaded into RBCEVs using REG1 following the manufacturer's protocol. 5 × 10^4^ MOLM13 cells were seeded in 48‐well plates 8 h in advance followed by incubation with 30 μg FAM ASO loaded CD33‐targeting/non‐targeting RBCEVs for 1 h at 37°C. After 2 washes with cold FACS buffer, the cells were analysed using flow cytometry.

### 2.9 Cell proliferation and viability assay

Cell proliferation and viability assays were measured with the Cell Counting kit‐8 (CCK‐8, Biosharp, China) following the manufacturer's instructions. For cell proliferation assay, MOLM13 cells were seeded at 1x10^4^ cells per well in 96‐well plates and treated with 15 μg FLT3‐ITD ASO or miR‐125b ASO or NC ASO‐loaded RBCEVs. At different time points (day 0, 1, 2, 3, 4), the CCK‐8 reagent was added and incubated for 2 h at 37°C. The absorbance at 450 nm was measured using a Synergy H1 microplate reader (BioTek, USA). For cell viability assay, MOLM13 cells were seeded at 3 × 10^4^ cells per well in 96‐well plates and treated with 15 μg ASO loaded RBCEVs for 72 h followed by CCK‐8 assay.

### 2.10 Cell apoptosis assay

Cell apoptosis was detected using Annexin V‐APC and Propodium Iodide (PI) staining (Biolegend, USA) according to the manufacturer's instructions. After treating MOLM13 cells with 15 μg ASO‐loaded RBCEVs for 72 h, the cells were washed twice with cold PBS and then resuspended in 100 μl Annexin V binding buffer. 5 μl of APC Annexin V and 10 μl of PI were added into cells followed by gentle vortexing of the cells and incubation for 15 min at room temperature in the dark. Before flow cytometry analysis, 400 μl of Annexin V binding buffer was added to each tube, and the samples were analysed using flow cytometry within 1 h.

### 2.11 Flow cytometry analysis

To determine the expression of surface protein, 1 × 10^5^ MOLM13 and CEM cells were washed 2 times with cold PBS and resuspended in 100 μl FACS buffer (PBS with 2% fetal bovine serum). 1 μl anti‐human CD33‐FITC antibody was added into above cells followed by an incubation of 30 min at 4°C in the dark. After 2 washes with FACS buffer, the cells were analysed by flow cytometry (CytoFLEX S system, Beckman Coulter, USA). The cell populations were first gated by FSC‐A and SSC‐A plot to exclude the debris and dead cells followed by using FSC‐width and FSC‐height to gate single cells. FITC channel was subsequently used to identify GFP‐positive population that indicated CD33‐positive cells.

For in vivo GFP^+^ leukaemia cells analysis, bone marrow from mouse femur or tibiae was collected by flushing the marrow cavities with FACS buffer using a syringe with 25G needle and filtering through 70 μm strainers. Liver tissues were cut into small pieces and homogenized in FACS buffer followed by filtering through 70 μm strainers. Splenic cells were isolated by gentle pressure‐dissociation of the spleen in FACS buffer and then filtered through 70 μm strainers. Peripheral blood was collected in EDTA‐treated tubes. After centrifuging, the above cell suspensions from the bone marrow, liver, spleen, and peripheral blood, RBC lysate buffer (ACK lysis buffer, Thermo Fisher Scientific) was added with incubation on ice for 5 min. 5 ml DMEM containing 10% FBS was then added into the mixtures to stop lysis. The cells were washed once with cold PBS and resuspended in FACS buffer. Flow cytometry and gate strategy were set up as described above. In the analysis of GFP^+^ leukaemia cells, the live cells were further gated from the single cells according to SYTOX™ blue negative staining (PB450 channel). The CytoFLEX LX, CytoFLEX S, or CytoFLEX system (Beckman Coulter, USA) were used for flow cytometry analysis, and the resulting FCS files were further analysed using FlowJo V10 software (FlowJo, USA).

### 2.12 Single EV flow cytometry

To determine the conjugation efficiency of RBCEVs with antibodies at single‐particle level, the NanoFCM system (NanoFCM, United Kingdom) was used for Single EV flow cytometry analysis. CD33 or IgG control antibody‐conjugated RBCEVs were stained with Alexa Fluor® 488 (AF488) AffiniPure donkey anti‐mouse IgG (Jackson ImmunoResearch, USA). RBCEVs were washed twice with filtered PBS at 21,000× *g* for 30 min, and samples were diluted 10,000‐fold before running on the NanoFCM machine. According to the manufacturer's protocol, we set the laser power at 8 mW, the SS decay at 10%, and the sampling pressure at 1.0 kPa before acquisition and recording.

### 2.13 Western blot analysis

Antibody‐conjugated RBCEVs were lysed with RIPA buffer (Thermo Fisher Scientific) containing protease inhibitors (Biotool) for 15 min on ice. Total cell lysates were extracted by incubating with RIPA buffer supplemented with protease inhibitors for 30 min on ice. A total of 100 μg protein from RBCEVs and 30 μg protein from cell lysates were loaded in 10% polyacrylamide gels along with a protein ladder (Precision Plus Protein™ Kaleidoscope, Bio‐Rad), followed by a transfer to Immobilon‐P polyvinylidene difluoride membrane (Merck Millipore). The membrane was blocked with 5% milk in 1× Tris‐buffered saline containing 0.1% Tween‐20 (TBS‐T) at room temperature for 2 h followed by an incubation with primary antibodies: rabbit anti‐FLT3 (CST), mouse anti‐GAPDH (A01020, Abbkine, USA), Pierce™ High Sensitivity Streptavidin‐HRP (Thermo Fisher Scientific) overnight at 4°C. We washed the blots 3 times with TBS‐T, and then incubated them with HRP‐conjugated anti‐mouse and anti‐rabbit secondary antibodies (Santa Cruz, USA) for 1 h at room temperature. The blots were imaged using the Bio‐Rad Chemidoc gel documentation system.

### 2.14 RNA extraction and RT‐qPCR

Peripheral blood samples were collected from 14 AML patients with informed consents at Queen Mary Hospital, Hong Kong. Total peripheral blood mononuclear cells were purified according to a previous protocol.^21^ The samples were confirmed to have at least 38% of leukaemia blasts. Total RNA extraction was carried out using TRIzol (ThermoFisher) based on the manufacturer's instructions. The quality and quantity of RNA samples were evaluated by NanoDrop analysis (ThermoFisher) and agarose gel electrophoresis. RNA samples were reverse transcribed into cDNAs using a reverse transcription kit (ThermoFisher) according to the manufacturer's protocol. Taqman® miRNA assays (ThermoFisher) were used to quantify the levels of miR‐125a and miR‐125b, and U6b RNA was used as the internal control. Ssofast Green qPCR kit (Bio‐Rad) was used to quantify the levels of FLT3‐ITD mRNAs, relative to the level of *GAPDH*. VIIA(TM) 7 System (Applied Biosystems, USA) was used to process all qPCR reactions.

### 2.15 Animal experiments

**FIGURE 1:**
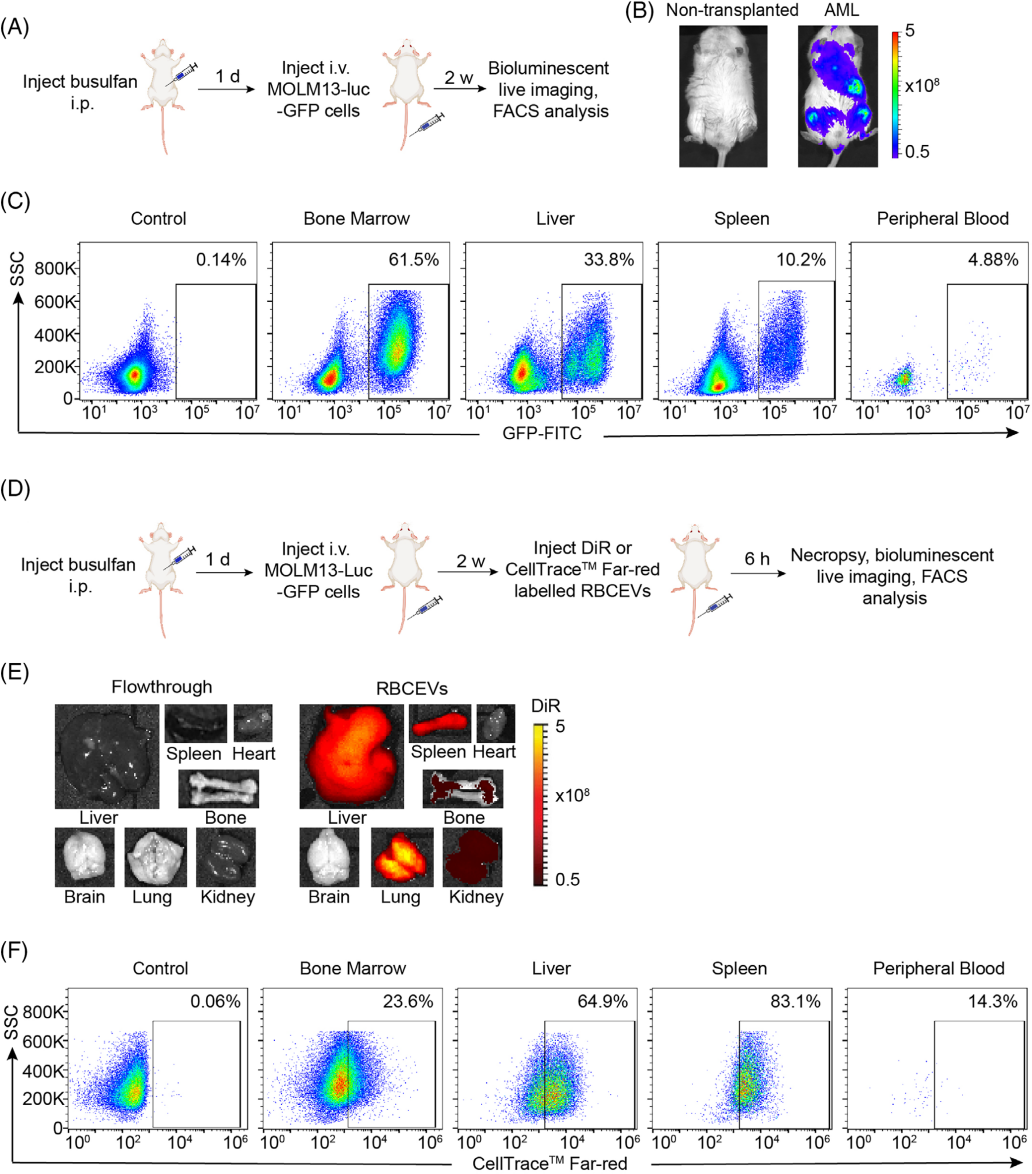
RBCEVs are taken up efficiently by leukaemia cells in xenografted mice. (A) Experimental schema of constructing AML xenograft model in NSG mice. (B) Bioluminescent imaging of non‐transplanted and AML‐xenografted mice. Colour scale indicates bioluminescent signals (photon/second). (C) Flow cytometric analysis of MOLM13‐Luc‐GFP cells in xenograft model, indicating the accumulation of leukaemia cells in the bone marrow, liver, spleen and peripheral blood. Samples were collected from NSG mice 14 days after i.v. injection of 5 × 10^5^ MOLM13 cells. (D) Experimental schema for determination of the biodistribution and uptake of RBCEVs in AML xenograft mice, flowthrough was the last wash of DiR‐labelled RBCEVs through SEC column. (E) In vivo fluorescent imaging analysis of DiR‐labelled RBCEVs in various organs of NSG mice administered by i.v. injection. Pseudocolors represent the DiR fluorescent intensity (photon/second). (F) Flow cytometric analysis of CellTrace™ Far‐red‐labelled RBCEVs taken up by GFP‐positive leukaemia cells, gated based on GFP expression, in the bone marrow, liver, spleen and peripheral blood of the EV‐injected mice

All animal experiments were performed following the protocols approved by the Institutional Animal Care and Use Committee at National University of Singapore (NUS) and the Animal Ethics Committees at City University of Hong Kong. NOD/SCID/IL2RΥgnull (NSG) mice and NSG‐SGM3 mice were provided by the Jackson Laboratory (USA) and bred in animal facilities of NUS and the City University of Hong Kong. The timelines of animal experiments are illustrated in the figures (Figures 1A,D, 3A, 4F, 5D, 6E, and 7E).

### 2.16 Establishment of leukaemia‐xenografted mice

For the MOLM13‐Luc‐GFP‐xenografted model, NSG or NSG‐SGM3 mice aged 7–8 weeks old were pre‐conditioned with Busulfan (Santa Cruz, 20 mg/kg) by tail vein injection. After 24 h, 5 × 10^5^ Luciferase‐GFP‐labelled MOLM13 cells were injected in the tail vein of the above‐conditioned mice. Around 1 week later, the mice were given an i.p. injection of D‐luciferin (150 mg/kg) to detect leukaemia signals based on bioluminescence using the IVIS Lumina III system (PerkinElmer, USA). The presence of bioluminescent signal in the bone marrow indicated the successfully engrafted AML mouse model.

For the patient‐derived xenograft (PDX) model, primary leukaemia cells were collected from AML patients with informed consent. The AML patient cells were injected into NSG mice via tail vein at a dose of 5 × 10^6^ cells/mouse. Mouse peripheral blood was collected via tail vein weekly to measure the percentage of human CD45^+^CD33^+^cells with FACS analysis. 8 to 12 weeks later, the animals were euthanized and the organs (bone marrow, liver, spleen, and peripheral blood) were harvested for FACS analysis to confirm the successful engraftment and following passage of PDX mice for treatment.

### 2.17 Biodistribution and uptake of RBCEVs in AML xenograft model

To determine the RBCEVs biodistribution, preconditioned MOLM13‐Luc‐GFP‐xenografted NSG mice with 2‐week‐old leukaemia were injected with 200 μg RBCEVs labelled with DiR (1,1′‐Dioctadecyl‐3,3,3′,3′‐Tetramethylindotricarbocyanine Iodide) and flowthrough control through tail vein injection. After 6 h, the mice were euthanized and the organs were harvested to detect DiR signals by the IVIS Lumina III system. To detect the uptake of RBCEVs by leukaemia cells, AML‐xenografted mice with 2‐week‐old leukaemia were intravenously injected with 1 mg CellTrace™ Far‐red‐labelled RBCEVs through the tail vein. After 6 h, mice were sacrificed and the organs were harvested as described above. CellTrace™ Far‐red fluorescence signal was measured in the leukaemia cells residing in the organs using flow cytometric analysis.

### 2.18 In vivo delivery of ASO‐loaded RBCEVs in AML‐xenografted mice

For the treatment of MOLM13‐Luc‐GFP‐xenografted model, successfully xenografted mice were randomized into control and treatment groups which were then intravenously injected with 40 mg/kg ASO‐loaded RBCEVs every 2 days. Every three days, the leukaemia signal was monitored by bioluminescent imaging using the IVIS Lumina III system as described above. At the endpoint of treatment, the mice were euthanized and the organs (blood, spleen, and liver) were harvested for FACS analysis or histopathology analysis.

For the treatment of patient‐derived xenograft (PDX) model, once the establishment of PDX mice which were detected with more than ~1% of human CD45^+^CD33^+^ in the peripheral blood, mice were randomized into control and treatment groups. PDX mice were subsequently injected with 40 mg/kg ASO‐loaded RBCEVs every 2 days through the tail vein. At the endpoint (4 weeks from the beginning of ASO‐loaded RBCEV treatment), when the control mice showed a symptom of weakness or death, all the mice were euthanized, and the organs were harvested for flow cytometric or histopathology analysis.

### 2.19 Histopathology analysis

Mouse spleens were fixed in 10% buffered formalin (ThermoFisher Scientific) overnight at room temperature, and processed with a tissue processor (Leica TP 1020, Germany): 70%, 95%, and 100% alcohol at 37°C, 3 baths of xylene (ThermoFisher Scientific) at 37°C and 3 baths of paraffin (ThermoFisher Scientific) at 62°C. Samples were cut into 4 μm sections using a microtome (Leica RM2135). The sections were dried at 37°C in an incubator, deparaffinized in xylene, rehydrated in ethanol of decreasing concentration followed by distilled water. The sections were stained in Haematoxylin Solution (Mayer's, Modified) (Abcam, Cat no. ab220365), rinsed in tap water, 2 quick dips in 2% ammonia, rinsed in tap water, rinsed in 70% ethanol (10 dips), counterstained with Shandon Eosin Y for 1 min, rinsed in ethanol of increasing concentration and finally rinsed in Xylene. The slides were mounted using DPX mounting medium (Sigma‐Aldrich, Cat No. 06522). Images were taken using an Invitrogen EVOS™ M7000 Imaging System in a blinded manner.

### 2.20 Primer sequences

*FLT3‐ITD* Forward: 5′‐AGAATATGAATTTGATTTCAGAG‐3′.

*FLT3‐ITD* Reverse: 5′‐CGGCAACCTGGATTGAGACT‐3′.

*FLT3* Forward: 5′‐GGTGACCGGCTCCTCAGATA‐3′.

*FLT3* Reverse: 5′‐GATCATATTCATATTCCCTT‐3′.

*GAPDH* Forward: 5′‐GGAGCGAGATCCCTCCAAAAT‐3′.

*GAPDH* Reverse: 5′‐GGCTGTTGTCATACTTCTCATGG‐3′.

### 2.21 Statistical analysis

Student's one‐tailed t‐tests were calculated using GraphPad Prism 8. Two‐way ANOVA was used for statistical analysis between multiple groups of treatments, also computed using GraphPad Prism 8. *p*‐values less than .05 were considered significant. Data are presented as mean and standard error of the mean (SEM).

## 3 RESULTS

### 3.1 RBCEVs are taken up efficiently by leukaemia cells in xenografted mice

To generate an in vivo model of AML, we injected luciferase‐GFP‐labelled MOLM13 cells (MOLM13‐Luc‐GFP) into the tail vein of busulfan‐conditioned NSG mice (Figure 1A). After 2 weeks, bioluminescent imaging indicated the successful engraftment of MOLM13‐Luc‐GFP cells in NSG mice (Figure 1B). GFP‐positive leukaemia cells were also detected in the bone marrow, liver, spleen, and peripheral blood of NSG mice using flow cytometric analysis (Figure 1C).

RBCEVs were purified as described in our previous study,^19^ with a typical cup‐shaped morphology and appeared intact under a transmission electron microscope (TEM), and an average diameter of ~160 nm determined by a Nanosight particle analyser (Figure S1A,B). To determine the biodistribution and cellular uptake of RBCEVs in AML‐xenografted mice, DiR or CellTrace™ Far‐red‐labelled RBCEVs were systemically injected into xenografted mice through the tail vein (Figure 1D). Six hours after injection of DiR‐labelled RBCEVs, in vivo imaging system (IVIS) analysis showed bright DiR signals mainly accumulated in the liver, lung, spleen, kidney, and bone. The flowthrough was the last wash of DiR‐labelled RBCEVs, that was the supernatant from the round of centrifugation after the size exclusion chromatography (SEC). This flowthrough control was used to remove the effect of background caused by leftover unbound dyes (Figure 1E). To analyse the uptake of RBCEVs by leukaemia cells in vivo, CellTrace™ Far‐red‐labelled RBCEVs were injected into MOLM13‐Luc‐GFP‐xenografted mice. After 6 h, flow cytometric analysis was used to detect the accumulation of RBCEVs by GFP‐positive leukaemia cells of the liver, lung, spleen, and peripheral blood (Figure 1F). FACS analysis showed that over 50% of leukaemic cells in the liver and spleen took up RBCEVs while leukaemic cells residing in the bone marrow and in peripheral blood showed lower levels of uptake.

### 3.2 Conjugation of RBCEVs with CD33‐targeting antibody promotes their uptake by CD33‐positive cells

While our data demonstrated that RBCEVs could be taken up by leukaemic cells, we observed that they also accumulated in non‐cancer cells. To increase the uptake specificity of RBCEVs, we sought to conjugate RBCEVs with an antibody that targets a surface marker overexpressed on cancer cells. CD33 is a member of the sialic acid‐binding immunoglobulin‐like lectin family, which is highly expressed on the majority of AML cells and is considered as a common leukaemia biomarker.^22^

**FIGURE 2:**
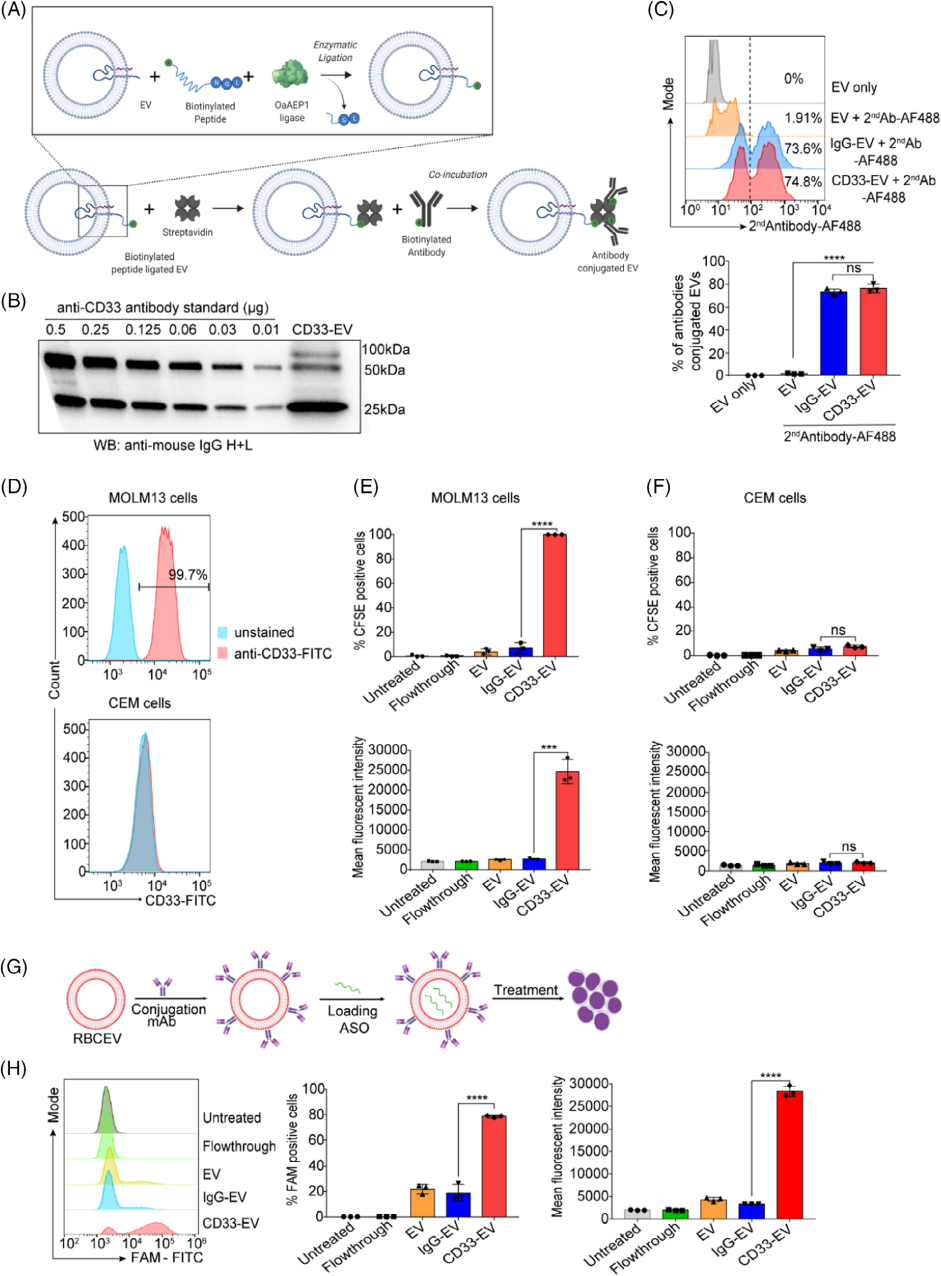
Conjugation of RBCEVs with anti‐CD33 antibody enables targeted delivery of RNA cargo to leukaemia cells (A) Outline of the streptavidin‐mediated approach for antibody conjugation following enzymatic ligation of a biotinylated peptide. (B) Western blot analysis of anti‐CD33 antibody conjugated to RBCEVs compared to a serial dilution of biotinylated anti‐human CD33 monoclonal antibody. The blot was probed with anti‐mouse IgG heavy and light chain (H + L). (C) Single‐EV flow cytometric analysis (NanoFCM) of monoclonal antibody conjugation on RBCEVs using the streptavidin‐mediated conjugation method. AF488‐conjugated anti‐mouse secondary antibody was used to detect the anti‐CD33 and IgG isotype control monoclonal antibody, CD33‐EV: anti‐CD33 antibody‐conjugated RBCEVs, IgG‐EV: isotype monoclonal antibody‐conjugated RBCEVs. (D) Flow cytometry analysis of CD33 expression on AML MOLM13 cells and leukaemic lymphoid CEM cells. (E, F) Flow cytometric analysis of CFSE indicating the uptake of CFSE‐labelled RBCEVs conjugated with CD33 mAb by CD33‐positive MOLM13 cells and CD33‐negative CEM cells, presented as the percentage and mean fluorescent intensity of CFSE. (G) Workflow for antibody conjugation of RBCEVs and subsequent ASO loading. (H) Flow cytometry analysis of FAM fluorescence reflecting the delivery of a FAM‐conjugated ASO (FAM ASO) to MOLM13 cells by CD33‐targeting or non‐targeting RBCEVs. The graphs present the mean ± SEM. Student's one‐tailed *t*‐test: ns, not significant, ***p < .001, ****p < .0001

Biotinylated anti‐human CD33 monoclonal antibody was conjugated onto RBCEVs surface through enzymatic ligation of a biotinylated peptide and the streptavidin‐biotin approach. The OaAEP1 protein ligase was initially used to mediate the ligation of a biotinylated peptide (B‐TL5) with RBCEVs. Following this, biotinylated anti‐CD33 antibody was conjugated to the biotin probe on the peptide through a streptavidin (SA) linker. Streptavidin is a tetramer containing four distinct biotin‐binding pockets and thus facilitates this conjugation (Figure 2A). Western blot showed 2 bands of the biotinylated anti‐CD33 antibody at ∼25 and 50 kDa, and anti‐CD33 antibody‐conjugated RBCEVs (CD33‐EV) displayed 3 prominent bands at ∼25, 50 and 100 kDa, detected using an anti‐mouse IgG(H + L) antibody (Figure 2B). Two bands at ∼25 and 50 kDa were also observed using streptavidin‐HRP for detection of biotinylated anti‐CD33 antibody; however, much more immunoglobulin heavy chain (IgH) was modified by biotin than Immunoglobulin light chain (IgL) (Figure S2A). So, there is higher chance of IgH binding to streptavidin to form a complex at ~100 kDa. To estimate the antibody conjugation efficiency at single‐RBCEV level, a NanoFCM system was used to detect RBCEVs coated with biotinylated anti‐CD33 antibody and isotype monoclonal antibody (IgG‐EV) following staining with an Alexa Fluor 488‐conjugated anti‐mouse IgG. The flow cytometric analysis indicated that ~74% of RBCEVs were successfully conjugated with the CD33 monoclonal antibody as displayed by the clear shift of the EV population to the right of the histogram (Figure 2C). To test the uptake specificity of anti‐CD33 antibody‐conjugated RBCEVs, we sought to identify the suitable cell line models for in vitro study. Flow cytometric analysis indicated that human AML MOLM13 cells overexpressed CD33 on their surface while acute lymphoblastic leukaemia CEM cells were negative for CD33 (Figure 2D). To determine the cellular uptake of CD33 targeting RBCEVs, we labelled CD33‐targeting and non‐targeting RBCEVs with CFSE dye before incubating them with leukaemia cells. Isotype antibody‐conjugated RBCEVs were used as a negative control to ensure that any changes in uptake were a result of specific interactions between the CD33‐binding domain of the antibody and not due to the conjugation process. As judged by the percentage of CFSE‐positive cells and mean fluorescent intensity of intracellular CFSE signals, conjugation of anti‐CD33 antibody significantly increased the uptake of RBCEVs by CD33‐positive MOLM13 cells, while non‐targeted RBCEVs were taken up at lower levels (Figure 2E). Moreover, low cellular uptake of CD33‐targeting and non‐targeted RBCEVs was seen in CD33‐negative CEM cells with no significant difference between the groups (Figure 2F). We subsequently used the CD33‐targeting RBCEVs to deliver RNA payloads specifically to target AML cells. FAM‐conjugated negative control (NC) ASO was loaded into RBCEVs using REG1 followed by incubation with MOLM13 cells (Figure 2G). Flow cytometry analysis demonstrated that the conjugation of RBCEVs with anti‐CD33 antibody increased the accumulation of FAM ASO in CD33‐positive MOLM13 cells (Figure 2H).

### 3.3 CD33‐targeting RBCEVs show a greater accumulation in xenografted CD33‐positive leukaemia cells

**FIGURE 3:**
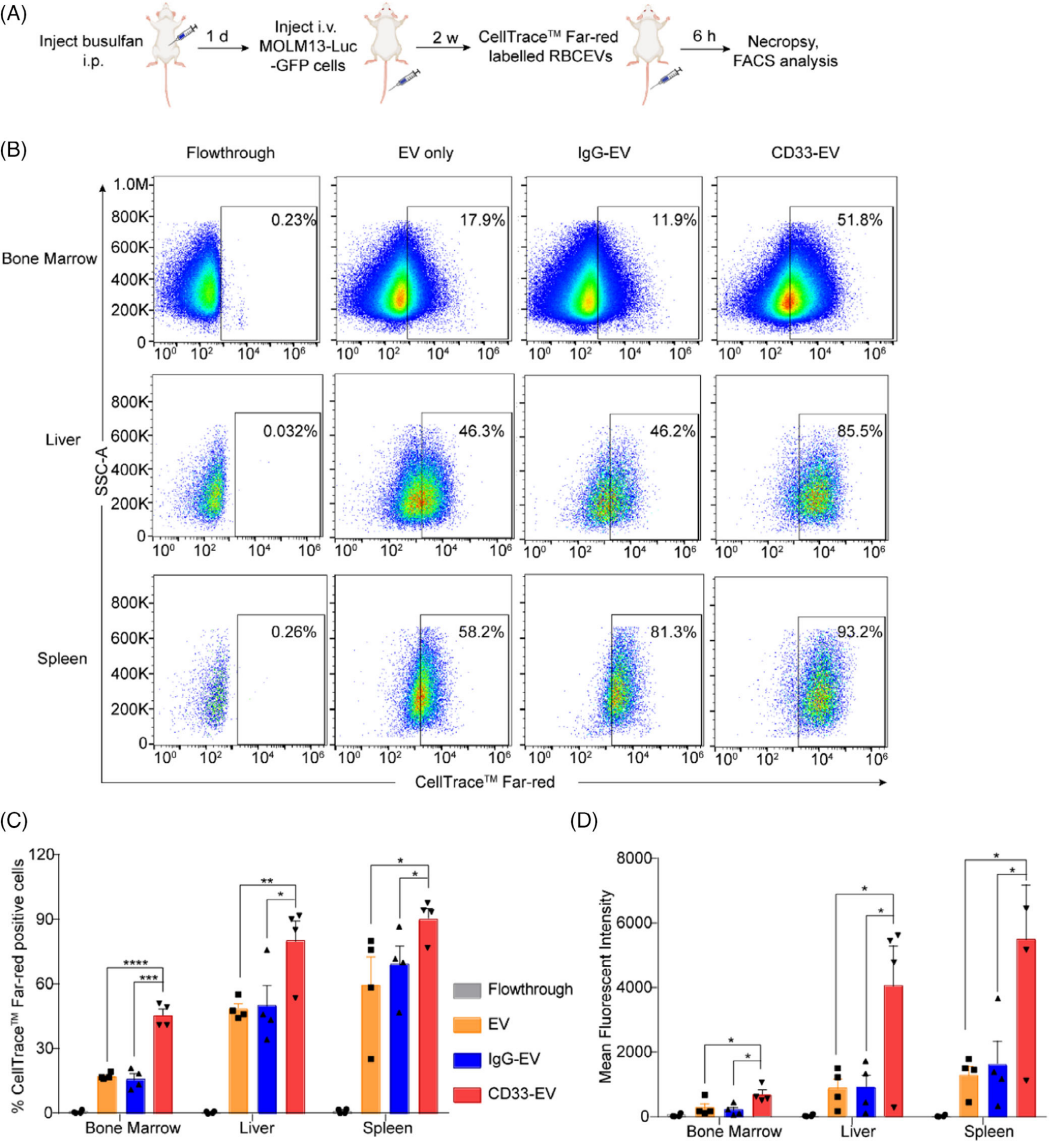
CD33‐targeting RBCEVs accumulate in xenografted CD33‐positive leukaemia cells at higher frequency than non‐targeting RBCEVs. (A) Workflow for determination of CD33‐targeting RBCEVs biodistribution in AML mice. (B) Flow cytometric analysis indicating the in vivo uptake of CellTrace™ Far‐red‐labelled RBCEVs by GFP‐positive leukaemia cells, gated based on GFP expression, in the bone marrow, liver, and spleen of the mice that were treated with control or anti‐CD33 mAb ligated RBCEVs. (C) Percentage of CellTrace™ Far‐red‐positive leukaemia cells in the bone marrow, liver, and spleen. (D) Mean fluorescent intensity of leukaemia cells that uptake CellTrace™ Far‐red ‐labelled RBCEVs. The graphs present the mean ± SEM. Student's one‐tailed *t*‐test: *p < .05, **p < .01, ***p < .001, ****p < .0001

After determining the enhanced accumulation of CD33‐targeting RBCEVs in target cells in vitro, we further verified the specific uptake of RBCEVs by leukaemia cells in vivo. AML‐xenografted mice were generated as described above (Figure 1A). When leukaemic bioluminescent signals became obvious and accumulated highly in the bone marrow, liver, and spleen of NSG mice, we labelled the CD33‐targeting/non‐targeting RBCEVs with CellTrace™ Far‐red dye and injected them into MOLM13‐Luc‐GFP cells xenografted mice (Figure 3A). After 6 h, we processed the bone marrow, liver, and spleen tissue and analysed the uptake of RBCEVs by GFP‐positive leukaemia cells of these tissues (Figure 3B). Interestingly, the conjugation of RBCEVs with anti‐CD33 antibody increased the percentage of CellTrace‐positive leukaemia cells from 14%–18% in the controls to 42%–52% in the CD33‐targeting EV treatment in the bone marrow, and from 40%–49% in the controls to 71%–89% in the CD33‐targeting EV treatment in the liver (Figure 3C). Similar increases in EV uptake were also observed in the spleen in the presence of CD33 targeting. The conjugation of RBCEVs with anti‐CD33 antibody led to more than a two‐fold increase in mean fluorescent intensity of CellTrace‐positive leukaemia cells in the bone marrow, liver, and spleen (Figure 3D). These data suggest that anti‐CD33 antibody can direct RBCEVs specifically into CD33‐positive leukaemia cells in vivo.

### 3.4 Systemic delivery of FLT3‐ITD ASO using RBCEVs suppresses AML progression

Mutations in FLT3 are the most prevalent in AML, and they are associated with an increased risk of relapse and decreased overall survival and disease‐free survival of AML patients.^23^ There are two common types of FLT3 mutations in AML: internal tandem duplication (ITD) mutations in the juxta‐membrane domain and point mutations in the tyrosine kinase domain (TKD).^24^ Currently, there is no targeted therapy available to treat patients with these mutations, except kinase inhibitors that act on all kinases, which are not specific to FLT3‐ITD, thus causing many side effects. Using ASOs against the FLT3‐ITD mutation can potentially provide a cancer‐specific treatment with minimal adverse effects.

**FIGURE 4:**
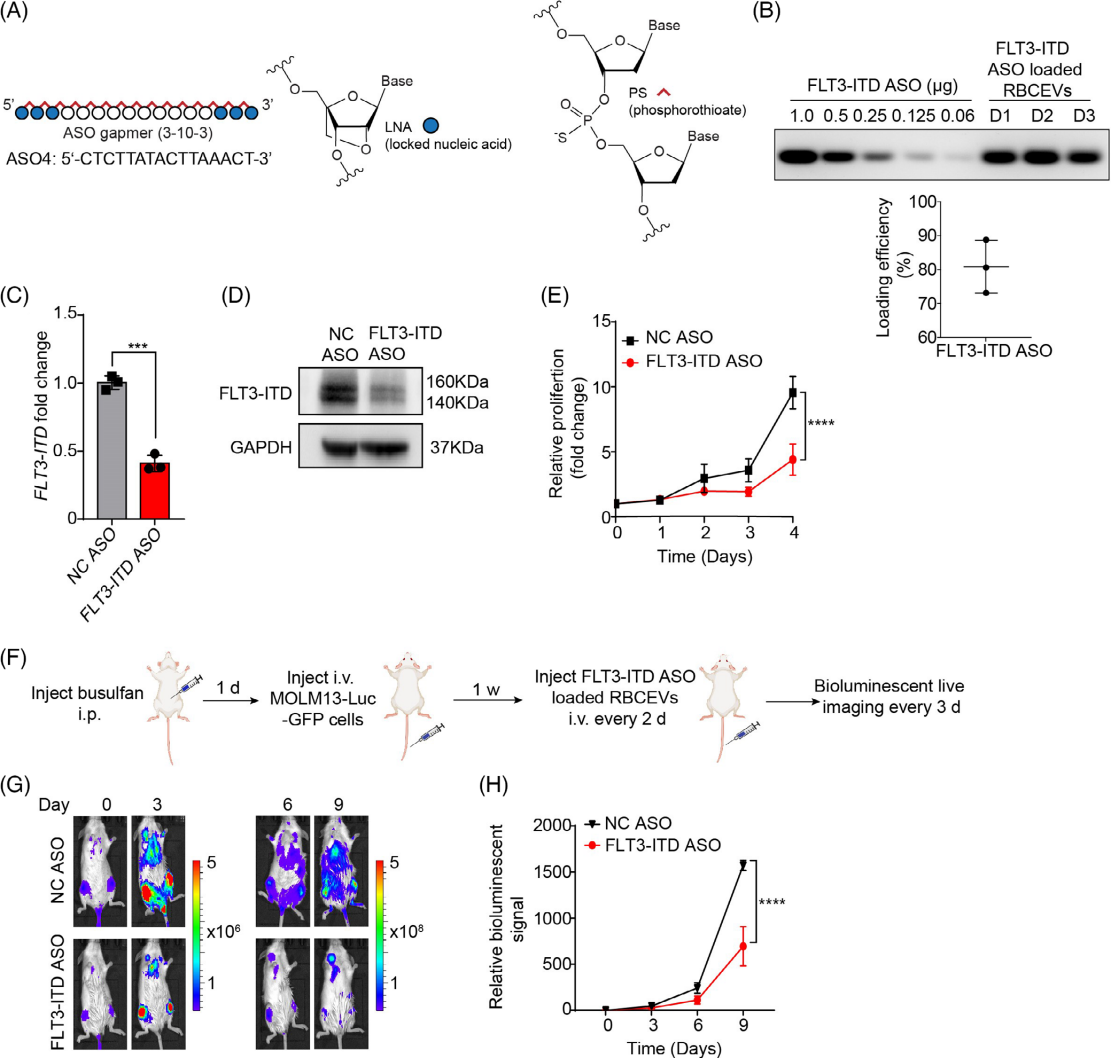
Systemic delivery of FLT3‐ITD ASO using RBCEVs suppresses AML progression. (A) Design of Locked Nucleic Acid (LNA) gapmer ASO against the repeated region of *FLT3‐ITD*. PS, phosphorothioate. (B) Gel electrophoresis separation of FLT3‐ITD ASO unloaded and loaded in RBCEVs from 3 different donors (D1, D2, and D3). (C) qPCR analysis of *FLT3‐ITD* expression in MOLM13 cells treated with negative control (NC)‐ASO or FLT3‐ITD ASO‐loaded RBCEVs, relative to *GAPDH* internal control. (D) Western blot analysis of FLT3‐ITD relative to GAPDH in leukaemia MOLM13 cells treated with NC ASO or FLT3‐ITD ASO. (E) Proliferation of MOLM13 cells upon FLT3‐ITD knockdown by FLT3‐ITD ASO loaded RBCEVs, determined using CCK‐8 assay. (F) Outline of AML xenografting and ASO delivery in NSG mice. (G) Representative bioluminescent images of NSG mice implanted with MOLM13‐luciferase‐GFP cells followed by ASOs‐loaded RBCEVs treatments. Pseudocolors represent bioluminescent total intensity (photon/second). (H) Leukaemia progression in mice quantified using the average bioluminescent signals following treatment with RBCEVs loaded with NC or FLT3‐ITD ASOs. The graphs present the mean ± SEM (*n* = 3 mice). Student's one‐tailed *t*‐test (E) and Two‐way ANOVA test (H): ***p < .001, ****p < .0001

We designed and screened a series of 16‐nucleotide (nt) antisense oligonucleotides (ASO) specifically against the repeated region of *FLT3‐ITD* (Figure S3A). The ASOs have a 3‐10‐3 gapmer configuration consisting of 3‐nt locked nucleic acid (LNA)‐modified ends flanking a central 10‐nt DNA segment, and they are further modified with phosphorothioate (PS) chemistry throughout the backbone. qPCR analysis found ASO4 targeting FLT3‐ITD to provide the best knockdown efficacy and specificity; hence, it was chosen for subsequent knockdown of *FLT3‐ITD* (Figures 4A and S3B,C). This FLT3‐ITD ASO was loaded into RBCEVs using REG1, and the loading efficiency was quantified using agarose gel electrophoresis. Comparison of ASO‐loaded RBCEVs against a standard curve of ASOs revealed that on average ~80% of ASO were loaded into RBCEVs (Figure 4B). After 24 h incubation of FLT3‐ITD ASO‐loaded RBCEVs with MOLM13 cells, which were known to have a heterozygous FLT3‐ITD mutation,^25^ a significant knockdown of *FLT3‐ITD* was detected using qPCR analysis (Figure 4C). Western blot analysis confirmed the decrease of FLT3‐ITD in MOLM13 cells treated with FLT3‐ITD ASO‐loaded RBCEVs (Figure 4D). Treating MOLM13 cells with FLT3‐ITD ASO‐loaded RBCEVs also significantly inhibited the proliferation of MOLM13 cells after 4 days of incubation, detected by CCK‐8 assay (Figure 4E).

We further tested the therapeutic efficacy of FLT3‐ITD ASO‐loaded RBCEVs in vivo. AML‐xenografted mice were generated by injecting MOLM13‐Luc‐GFP cells in the tail vein of NSG mice as described above. After 1 week when leukaemic bioluminescent signals became detectable, the tumour‐bearing mice were treated with FLT3‐ITD ASO‐loaded RBCEVs or negative control (NC) ASO‐loaded RBCEVs systemically (i.v.) every two days. Tumour burden was recorded by monitoring leukaemic bioluminescent signals every three days (Figure 4F). Mice treated with FLT3‐ITD ASO‐loaded RBCEVs had a much lower leukaemic burden compared to NC ASO‐loaded RBCEVs treated group, determined by the leukaemic bioluminescent signals during the ASO‐RBCEVs treatment (Figure 4G). Figure 4H showed the relative bioluminescent signals normalized to day 0, indicating that the FLT3‐ITD ASO‐loaded RBCEVs treatment significantly suppressed leukaemia progression.

### 3.5 CD33‐targeting RBCEVs enhance anti‐tumour efficacy of FLT3‐ITD ASO

**FIGURE 5:**
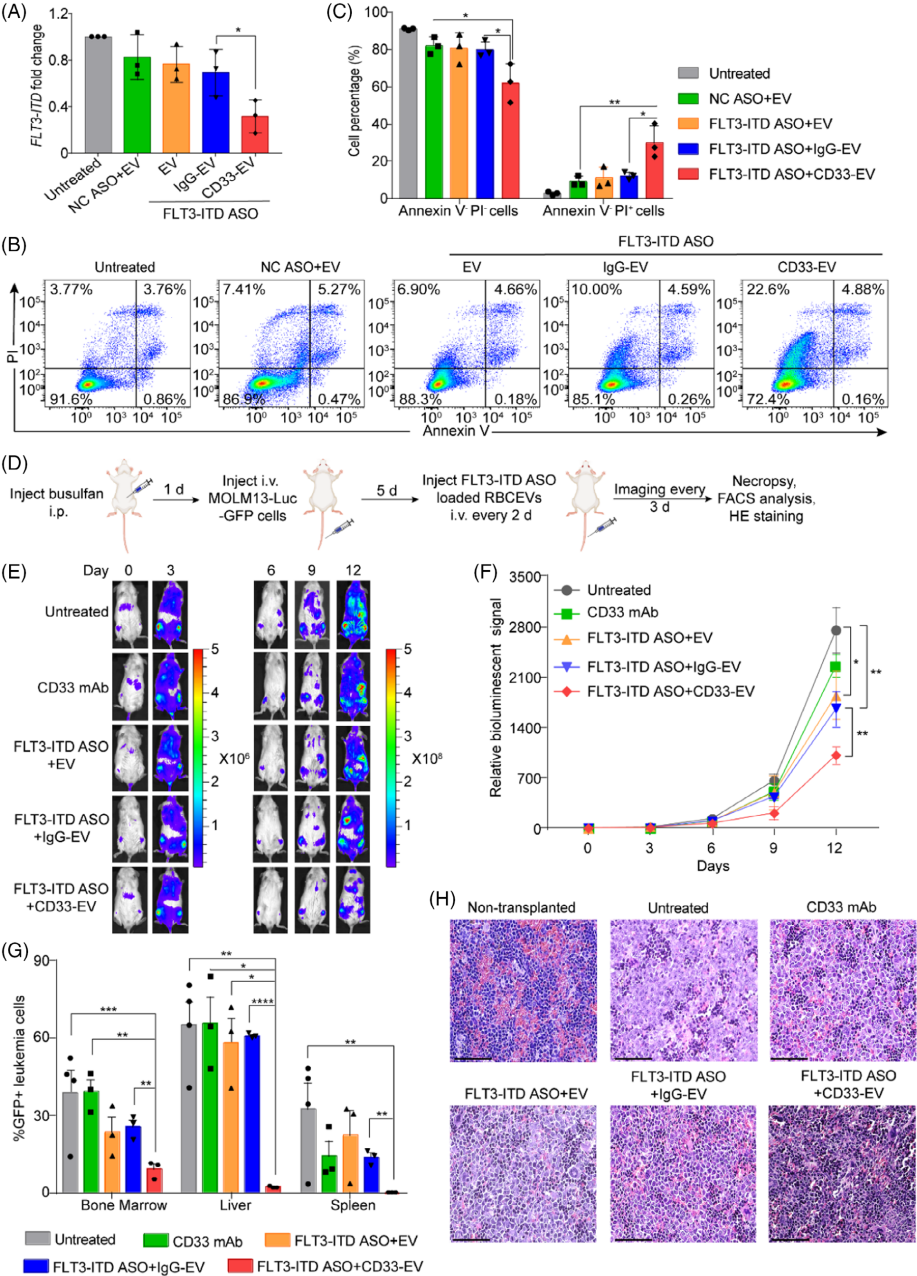
Conjugation of RBCEVs with anti‐CD33 antibody enhances the delivery of FLT3‐ITD ASO and its effect in suppressing leukaemia progression. (A) SYBR Green qRT‐PCR analysis of *FLT3‐ITD* in MOLM13 cells treated with FLT3‐ITD ASO‐loaded CD33/non‐targeting RBCEVs, presented as fold change relative to the untreated group and normalized to *GAPDH*. (B) Apoptosis of MOLM13 cells following a 72 h treatment with FLT3‐ITD‐ASO‐loaded RBCEVs with or without CD33 targeting, determined by Annexin V and PI staining. (C) Percentage of Annexin^−^PI^−^ viable cells and Annexin^−^PI^+^ necrotic cells among MOLM13 cells that were treated with FLT3‐ITD ASO‐loaded RBCEVs with or without CD33 targeting. (D) Outline of the treatment with FLT3‐ITD ASO‐loaded RBCEVs with or without CD33 targeting in leukaemia xenograft model. (E) Representative bioluminescent images of NSG mice implanted with MOLM13‐Luc‐GFP cells following RBCEV treatments. (F) Leukaemia progression in mice quantified using the average bioluminescent signals following treatment with FLT3‐ITD ASO‐loaded RBCEVs with or without CD33 targeting, relative to day 0. The graphs present the mean ± SEM (*n* = 4 mice). (G) Percentage of GFP‐positive leukaemia cells in the bone marrow, liver, and spleen. (H) Representative H&E staining images of the spleen sections from leukaemic mice treated with different conditions and non‐transplanted mouse. Scale bar, 75 μm. Student's one‐tailed *t*‐test and Two‐way ANOVA test (E): *p < .05, **p < .01, ***p < .001, ****p < .0001

CD33‐positive MOLM13 cells treated with FLT3‐ITD ASO‐loaded CD33‐targeting RBCEVs showed a significant decrease of *FLT3‐ITD* levels compared to the control groups (Figure 5A). Annexin V and PI staining was used to detect the viability of MOLM13 cells treated with FLT3‐ITD ASO‐loaded RBCEVs with or without CD33‐targeting. CD33 targeted RBCEVs loaded with FLT3‐ITD ASO significantly increased the percentage of necrotic MOLM13 cells (Annexin^−^PI^+^ cells) from 11%–13% in the controls to 25%–35% in the targeted EV‐CD33 treatment, while concurrently decreasing the percentage of viable cells (Annexin^−^PI^−^ cells) in targeted EV‐CD33 group (Figure 5B,C). To test the tumour‐suppressive efficacy of CD33‐targeting RBCEVs loaded FLT3‐ITD ASO in vivo, AML‐xenografted mice were generated by injecting MOLM13‐Luc‐GFP cells as described above (Figure 5D). Bioluminescent images were used to monitor leukaemia progression in the xenografted mice. On day 12, the leukaemic bioluminescent signal decreased significantly in mice treated with CD33‐targeting RBCEVs loaded FLT3‐ITD ASO compared to the other control groups (Figure 5E). Relative bioluminescent signals normalized to day 0 also showed that the CD33‐targeting RBCEVs loaded FLT3‐ITD ASO significantly suppressed AML progression (Figure 5F). The percentage of GFP‐positive leukaemia cells also reduced significantly in the bone marrow, liver, and spleen of the mice treated with CD33‐targeting RBCEVs loaded with FLT3‐ITD ASO as compared to the control groups (Figure 5G). Figure 5H showed the H&E staining of spleen sections, revealing much lesser infiltration of leukaemia cells in the spleen of FLT3‐ITD ASO‐loaded CD33‐targeting RBCEV treated mice compared to the control groups.

### 3.6 Systemic delivery of miR‐125b ASO loaded RBCEVs suppresses AML progression

**FIGURE 6:**
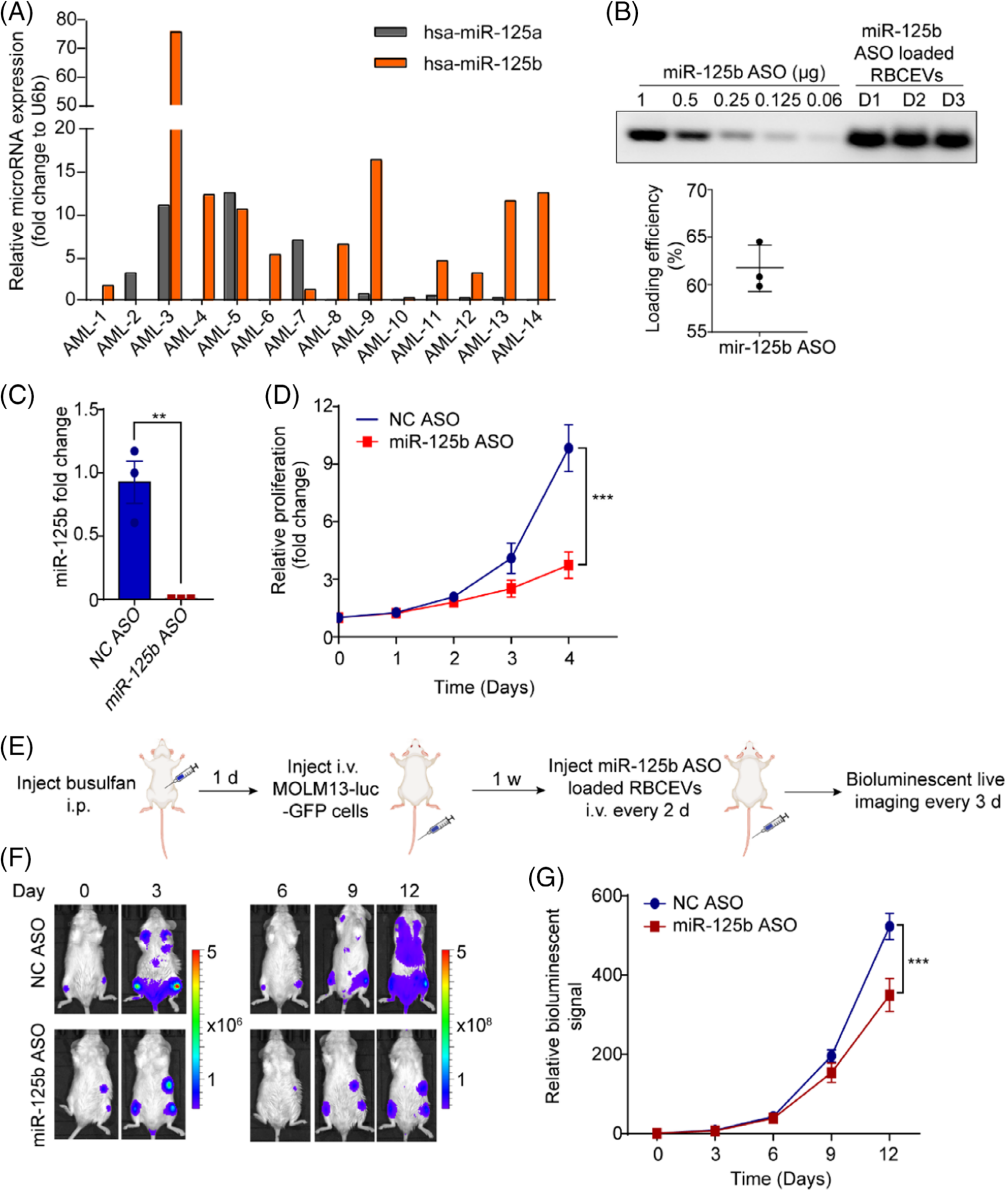
Systemic delivery of miR‐125b ASO using RBCEVs suppresses AML progression. (A) Expression of miR‐125b and miR‐125a in AML patient samples, determined by Taqman RT‐qPCR normalized to *U6b* RNA. (B) Gel electrophoresis separation of miR‐125b ASO unloaded and loaded in RBCEVs from 3 different donors (D1, D2, and D3). (C) Knockdown of miR‐125b in MOLM13 cells using NC ASO or miR‐125b ASO‐loaded RBCEVs, determined using qPCR as in (A). (D) Proliferation of MOLM13 cells upon miR‐125b knockdown by miR‐125b ASO‐loaded RBCEVs, determined using CCK‐8 proliferation assay. (E) Outline of the treatment with miR‐125b ASO‐loaded RBCEVs in MOLM13 leukaemia xenograft model. (F) Representative bioluminescent images of NSG mice implanted with MOLM13‐luciferase‐GFP cells and treated with ASO‐loaded RBCEVs. (G) Average bioluminescent signals in mice monitoring the development of leukaemia following treatment with NC or miR‐125b ASO loaded RBCEVs, normalized to the signals before the treatment started (day 0). The graphs present the mean ± SEM (*n* = 4 mice). Student's one‐tailed *t*‐test and Two‐way ANOVA test (G): ns, not significant, **p < .01, ***p < .001

Overexpression of miR‐125b is frequently observed in human AML.^26^ miR‐125b promotes AML cell expansion and suppresses apoptosis, thus enhancing the development of AML and drug resistance.^27^ Hence, suppression of miR‐125b by using ASOs could provide a novel therapeutic approach to AML treatment. High expression of miR‐125b was detected in 10 out of 14 AML patient samples at higher levels relative to the abundant house‐keeping *U6b* RNA, as quantified using TaqMan qRT‐PCR (Figure 6A). miR‐125a, the homologue of miR‐125b, was also overexpressed in 3 AML patients (Figure 6A).

We previously used an ASO with 2′‐O‐methyl modification to knockdown miR‐125b efficiently.^19^ However, our method of loading miR‐125b ASO into RBCEVs using electroporation resulted in aggregation of EVs. Since we have tested the new method for loading FLT3‐ITD using REG1 reagent and did not observe the aggregation problem, we sought to validate this method further with miR‐125b ASO. miR‐125b ASO was loaded into RBCEVs using REG1, and quantified utilizing agarose gel electrophoresis. Compared to a standard curve of free miR‐125b ASO, an average of ~60% of miR‐125b ASO was loaded into RBCEVs (Figure 6B). miR‐125b ASO‐loaded RBCEVs significantly decreased the expression of miR‐125b in MOLM13 cells (Figure 6C) and inhibited the growth of MOLM13 cells as measured by CCK‐8 assay (Figure 6D). To further test the therapeutic potential of miR‐125b ASO‐loaded RBCEVs in vivo, MOLM13‐Luc‐GFP‐xenografted mice were treated with miR‐125b ASO‐loaded RBCEVs or NC ASO‐loaded RBCEVs every 2 days. The tumour burden was recorded by monitoring leukaemic bioluminescent signals every 3 days (Figure 6E). The mice that were treated with miR‐125b‐ASO‐loaded RBCEVs developed leukaemia much more slowly than the control group (Figure. 6F,G).

### 3.7 CD33‐targeting RBCEVs enhance the delivery of miR‐125b ASO and its effect in suppressing leukaemia progression in patient‐derived AML xenografts

**FIGURE 7:**
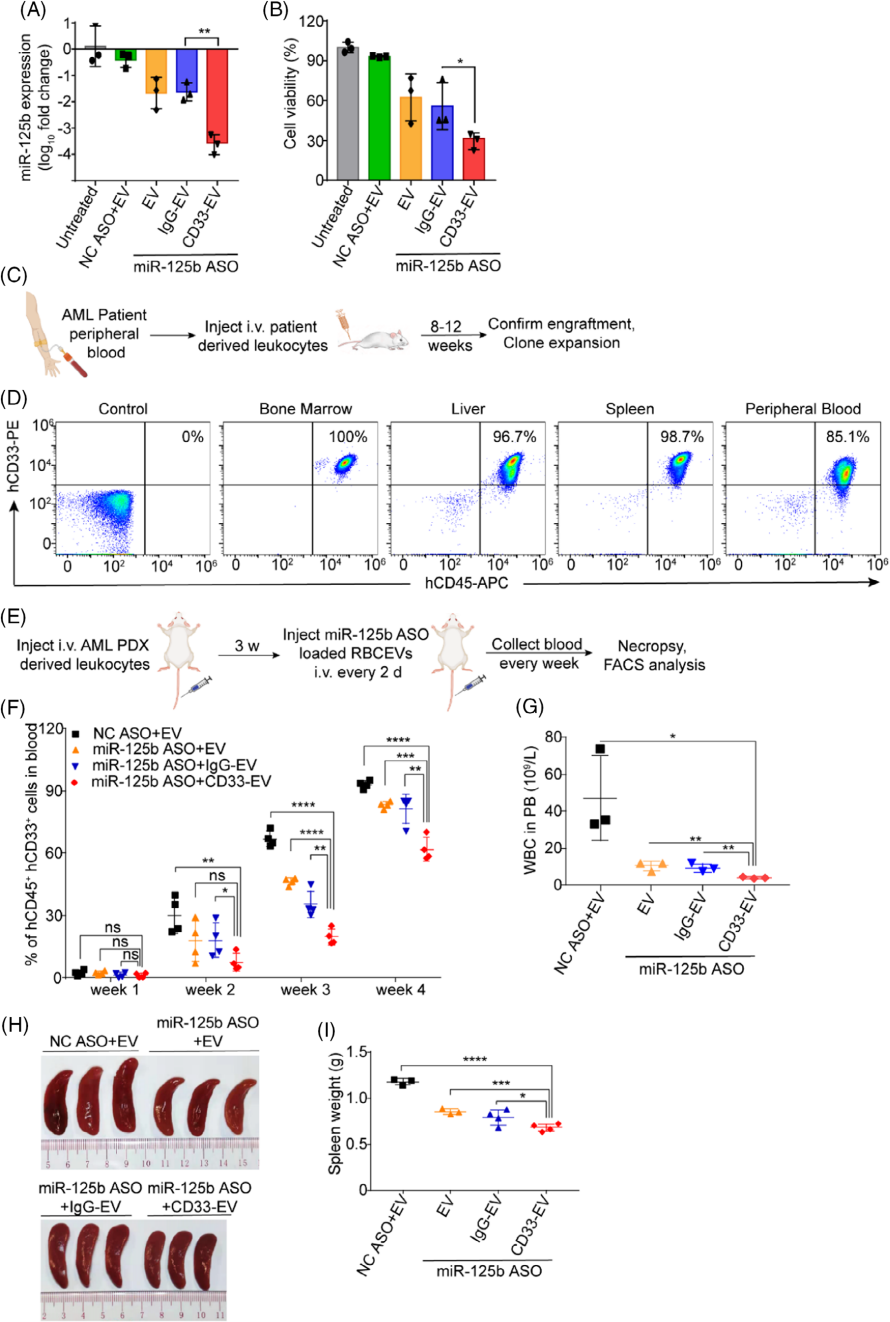
Conjugation of RBCEVs with anti‐CD33 antibody enhances the delivery of miR‐125b ASO loaded RBCEVs and its effect in suppressing leukaemia progression in patient derived AML xenografts. (A) Knockdown of miR‐125b in MOLM13 cells using NC ASO or miR‐125b ASO‐loaded RBCEVs, with or without CD33 targeting, presented as average log_10_ fold change relative to the untreated control, determined using Taqman RT‐qPCR, normalized to *U6b* RNA. (B) Viability of MOLM13 cells following treatment with miR‐125b ASO‐loaded RBCEVs with or without CD33 targeting, determined using CCK8 assay. (C) Experimental schema of AML PDX model. (D) Phenotype of PDX mice at late stage of AML, detecting the accumulation of human CD45^+^CD33^+^ leukaemia cells in the bone marrow, liver, spleen, and peripheral blood of PDX mice using flow cytometry analysis. (E) Outline of the treatment scheme with miR‐125b ASO loaded RBCEVs with or without CD33 targeting in PDX model. (F) Percentage of human CD45^+^CD33^+^ cells in the peripheral blood of PDX mice treated with miR‐125b ASO‐loaded RBCEVs with or without CD33 targeting. (G) White blood cells (WBC) count in mice peripheral blood (PB) on week 4. (H) Spleen size of PDX mice on week 4. (I) Weight of the spleens from PDX mice treated with miR‐125b ASO‐loaded RBCEVs with or without CD33 targeting. The graphs present the mean ± SEM. Student's one‐tailed *t*‐test: *p < .05, **p < .01, ***p < .001, ****p < .0001

CD33‐positive MOLM13 cells treated with miR‐125b ASO‐loaded CD33‐targeting RBCEVs showed more than 10‐fold decrease in miR‐125b levels compared to the control groups (Figure 7A). Moreover, CD33‐targeting RBCEVs loaded with miR‐125b ASO significantly reduced the viability of MOLM13 cells compared to the control groups (Figure 7B).

To test the therapeutic effect of miR‐125b ASO‐loaded CD33‐targeting RBCEVs in a preclinical model, we sought to establish AML patient‐derived xenograft (PDX) models using CD45^+^CD33^+^ peripheral blood cells from an AML patient with a high level of miR‐125b (Figure 7C). PDX models retain the genetic characteristics of their donor tumour and broadly represent the complex clinical tumour heterogeneity and molecular diversity of human cancers which is becoming the preferred preclinical model in oncology drug development.^28^, ^29^ Primary leukaemia cells were collected from AML patients with informed consent. The AML cells were injected into NSG mice at a dose of 5 million cells per mouse. Leukaemia development was monitored by detecting the percentage of human CD45^+^CD33^+^cells in mouse peripheral blood every week. Once more than 1% human CD45^+^CD33^+^cells were detected, the PDX was considered successful (Figure 7C). At the late stage of AML PDX, more than 80% of human leukaemia cells can be detected in mice peripheral blood and ~96% of human leukaemia cells can be observed in the mice's liver, spleen, and bone marrow (Figure 7D). After sufficiently expanding the PDX colony with ~1% human leukaemia cells in the peripheral blood, the mice were treated with miR‐125b ASO‐loaded CD33‐targeting/non‐targeting RBCEVs. The percentage of human AML cells in mouse peripheral blood was tested every week to monitor leukaemia progression (Figure 7E). Flow cytometry analysis revealed that mice treated with miR‐125b ASO‐loaded CD33‐targeting RBCEVs had much lower percentages of human leukaemia cells compared to control mice, indicating lower leukaemic burden and slower leukaemia progression (Figure 7F). Considering the white blood cells (WBC) count below 1.6 × 10^9^/L in NSG mice,^30^ the majority of WBC detected in mice peripheral blood are human leukaemia cells. Figure 7G showed decreased WBC count in the peripheral blood of mice treated with miR‐125b ASO‐loaded CD33‐targeting RBCEVs compared to the control groups, indicating lesser circulating human leukaemia cells in mouse blood. Furthermore, smaller spleens and lighter spleen weight were observed in mice treated with miR‐125b ASO‐loaded CD33‐targeting RBCEVs compared to their control groups (Figure 7H,I). Taken together, conjugation of RBCEVs with anti‐CD33 antibody significantly promotes the delivery of miR‐125b ASO‐loaded RBCEVs thus enhancing the suppression of leukaemia in patient‐derived AML xenografts.

## 4 DISCUSSION

At advanced stages, the systemic metastasis of AML poses great challenges to the treatment outcome and patient survival.^31^ Consistent genetic changes, prevalence of drug resistance phenotype, and off‐targeting effects of the drugs are among the several factors which contribute to the treatment failure.^32^ Exploration of new drug regimens has led to the growing emergence of RNA therapeutics for AML. Of particular note, small RNAs including antisense oligonucleotides (ASOs) hold a lot of promise in programmable therapies to target the diseased human genome. However, rapid clearance of small RNAs by the renal filtration system and circulating immune cells remains a major challenge limiting their clinical potential. In addition, delivery vehicles used to protect and deliver RNA molecules, i.e., viral vectors, lipoplexes (lipid‐based transfection reagents) and formulated nanoparticles are usually immunogenic and exert another layer of complexity. Here, we harness RBCEVs as a safe RNA‐drug delivery system,^19^ combined with surface modification of vesicles to specifically deliver the ASOs to target cells. We believe, this approach will accelerate the transformation of therapeutic ASOs to clinical application and pave a way for the better outcome of the treatment in leukaemia patients.

In our previous study, we demonstrated that RBCEVs could overcome many limitations of competitive technologies including the impossibility of scalability and toxicity.^19^ More recently, to confer the RBCEVs with specific tissue homing, we validated an enzymatic method for conjugation of targeting peptides and nanobodies onto RBCEVs, which is a gentle approach with minimized surface damage and instability of RBCEVs.^20^ Here, we further advanced our conjugation protocol and demonstrated its therapeutic potential as a versatile RNA‐drug delivery system.

The peptides and nanobodies against the antigen epitope present on the targeting cells require additional 3 amino acid sequences at their c‐terminal to mediate the ligation process. Considering the form of the targeting moieties used in the enzymatic method, there are certain limitations including the limited nanobody and peptide libraries, and high specificity and affinity nanobodies are not available against many cellular antigen epitopes. A feasible and promising alternative is antibodies, as they are readily available against mostly known antigens. In this study, we conjugated RBCEVs with antibodies through the enzymatic ligation of a biotinylated peptide and the streptavidin‐biotin system. This conjugation method takes advantage of the high affinity and specificity of the biotin‐streptavidin interaction, one of the strongest known non‐covalent interactions between a protein and its ligand while avoiding the disadvantage of surface damage caused by chemical modification during biotinylation of EV membrane. The antibody‐conjugated RBCEVs were effectively and selectively taken up by the target cells both in the in vitro and in vivo settings.

The surface‐modified RBCEVs can be utilized as a personalized medicine tool to deliver ASOs targeting different genetic aberrations in leukaemia. Here, we demonstrated the enhanced efficacy of RBCEVs to deliver ASOs against *FLT3‐ITD* and miR‐125b in AML cells. *FLT3* gene is found to be mutated in approximately 25% of AML, and 2%–4% of chronic myeloid leukaemia (CML) patients. Indeed, the mutation sequence in the FLT3‐ITD region varies among different patients. Hence, it requires sequencing of individuals to obtain the detailed *FLT3‐ITD* gene mapping before designing specific ASOs against the FLT3‐ITD mutations and thus providing a more personalized and effective treatment.^33^ miR‐125b is a well‐known oncogenic miRNA and its altered expression is reported in different subtypes of leukaemia.^26^ The high expression of miR‐125b in leukaemia patients is another cofounding factor that accelerates disease pathogenesis.^27^ It has been reported that miR‐125b is highly upregulated not only in patients with AML, CML, and acute lymphoblastic leukaemia (ALL) but also in children with Down syndrome—acute megakaryoblastic leukaemia due to the duplication of mir‐125b‐2 locus.^34^, ^35^ So miR‐125b‐ASO‐based treatment can benefit many leukaemia patients. Moreover, screening of patients with high expression of miR‐125b, along with FLT3‐ITD mutation will open several avenues to target both genes simultaneously and further expand the pivotal role of RBCEV‐delivered ASOs in personalized therapy. Considering that cancer is a complex and heterogeneous disease involving various molecular aberrations and the deregulation of multiple signal transduction pathways,^36^ combination therapy based on ASOs targeting different oncogenes may provide synergistic anti‐tumour effects.^37^, ^38^, ^39^ As *FLT3‐ITD* and miR‐125b are two common oncogenes in AML, combined treatments with ASOs targeting *FLT3‐ITD* and miR‐125b are expected to have synergistic therapeutic effects that are more potent than each ASO alone.

## AUTHOR CONTRIBUTIONS

C.H. designed and performed experiments, analyzed the data and wrote the manuscript. M.K.J. helped with the EV characterization and writing. E.Y.M.Y. helped with histopathology analysis. W.Z. helped with histopathology and writing. M.P. helped with the project design and paper writting. W.M.U. performed qPCR analysis of miR‐125 microRNAs in AML patients and helped with the writing. T.T.P. helped with mouse experiments and writting. K.W.L and N.V.T designed and synthesized FLT3‐ITD ASOs. A.Y.H.L., X.D. and Q.Z provided AML samples and funding. A.T.P. supervised the ASO generation. M.T.N.L. conceptionalized the project, obtained funding, provided training, supervised the study, analyzed the data and wrote the manuscript.

## CONFLICT OF INTEREST

Minh T. N. Le is the scientific cofounder and advisor of Carmine Therapeutics. Marco Pirisinu is an employee of Jotbody Ltd. Other authors do not have any conflict of interest.

## Supporting information


**Figure S1** Characterization of RBVEVs. (A) Transmission electron microscopy images of purified RBCEVs stained with uranyl acetate negative stain. Scale bar, 200 nm. (B) Nanoparticle tracking analysis of purified RBCEVs displaying the size distribution profile. Data was obtained using a Zetaviewer system
**Figure S2** Western blot analysis of biotinylated anti‐human CD33 antibody. (A) Western blot analysis of a serial dilution of biotinylated anti‐human CD33 monoclonal antibody. The blot was probed with streptavidin‐HRP
**Figure S3** Design and screening of FLT3‐ITD ASOs. (A) Detail sequences of 10 ASOs targeted FLT3‐ITD mutation of MOLM13 cells. Black colour sequences came from wild *FLT3* and Red colour sequences came from the ITD mutation. (B) Test and screening of FLT3‐ITD ASOs in MOLM13 cells at 24 h. qPCR analysis of *FLT3‐ITD* and *FLT3* expression in MOLM13 cells treated or untreated with FLT3‐ITD ASO loaded RBCEVs, relative to *GAPDH* internal control. (C)Test and screening of FLT3‐ITD ASOs in MOLM13 cells at 48 h following above protocol

## Data Availability

The authors declare that all data of article and Supplementary materials are available upon reasonable request.
