# Supplementary material for: CD33‐targeting extracellular vesicles deliver antisense oligonucleotides against FLT3‐ITD and miR‐125b for specific treatment of acute myeloid leukaemia
Source: Cell Prolif. 2022 Jul 18;55(9):e13255. doi: 10.1111/cpr.13255 (PMC9436904; doi:10.1111/cpr.13255)
Supplement: Supplementary file 1 — Figure S1 Characterization of RBVEVs. (A) Transmission electron microscopy images of purified RBCEVs stained with uranyl acetate negative stain. Scale bar, 200 nm. (B) Nanoparticle tracking analysis of purified RBCEVs displaying the size distribution profile. Data was obtained using a Zetaviewer system Figure S2 Western blot analysis of biotinylated anti‐human CD33 antibody. (A) Western blot analysis of a serial dilution of biotinylated anti‐human CD33 monoclonal antibody. The blot was probed with streptavidin‐HRP Figure S3 Design and screening of FLT3‐ITD ASOs. (A) Detail sequences of 10 ASOs targeted FLT3‐ITD mutation of MOLM13 cells. Black colour sequences came from wild FLT3 and Red colour sequences came from the ITD mutation. (B) Test and screening of FLT3‐ITD ASOs in MOLM13 cells at 24 h. qPCR analysis of FLT3‐ITD and FLT3 expression in MOLM13 cells treated or untreated with FLT3‐ITD ASO loaded RBCEVs, relative to GAPDH internal control. (C)Test and screening of FLT3‐ITD ASOs in MOLM13 cells at 48 h following above protocol [file CPR-55-e13255-s001.docx]

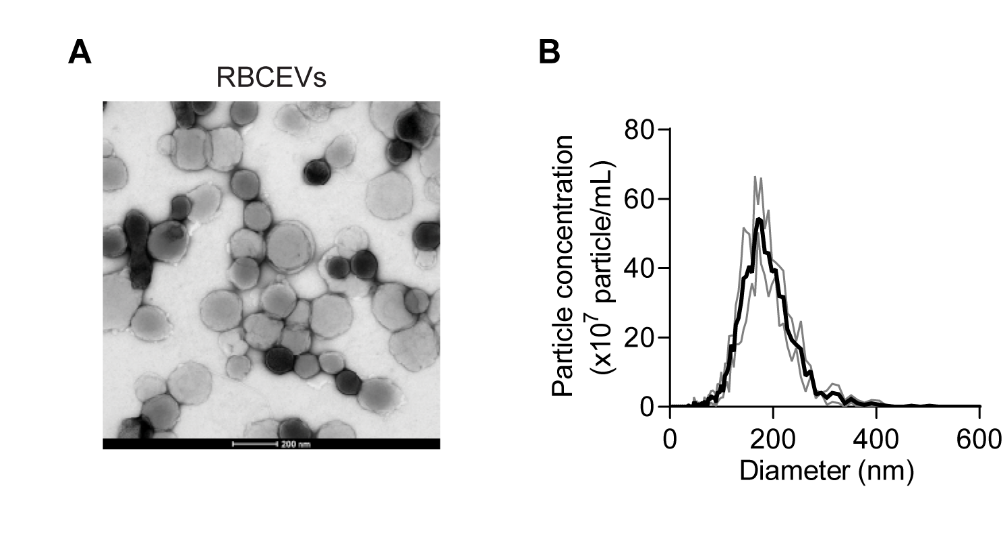


**Figure S1| Characterization of RBVEVs.** (A) Transmission electron microscopy images of purified RBCEVs stained with uranyl acetate negative stain. Scale bar, 200nm. (B) Nanoparticle tracking analysis of purified RBCEVs displaying the size distribution profile. Data was obtained using a Zetaviewer system.


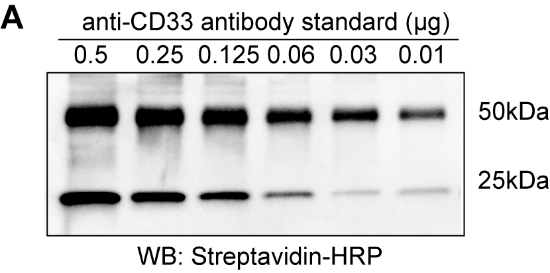


**Figure S2| Western blot analysis of biotinylated anti-human CD33 antibody.** (A) Western blot analysis of a serial dilution of biotinylated anti-human CD33 monoclonal antibody. The blot was probed with streptavidin-HRP.


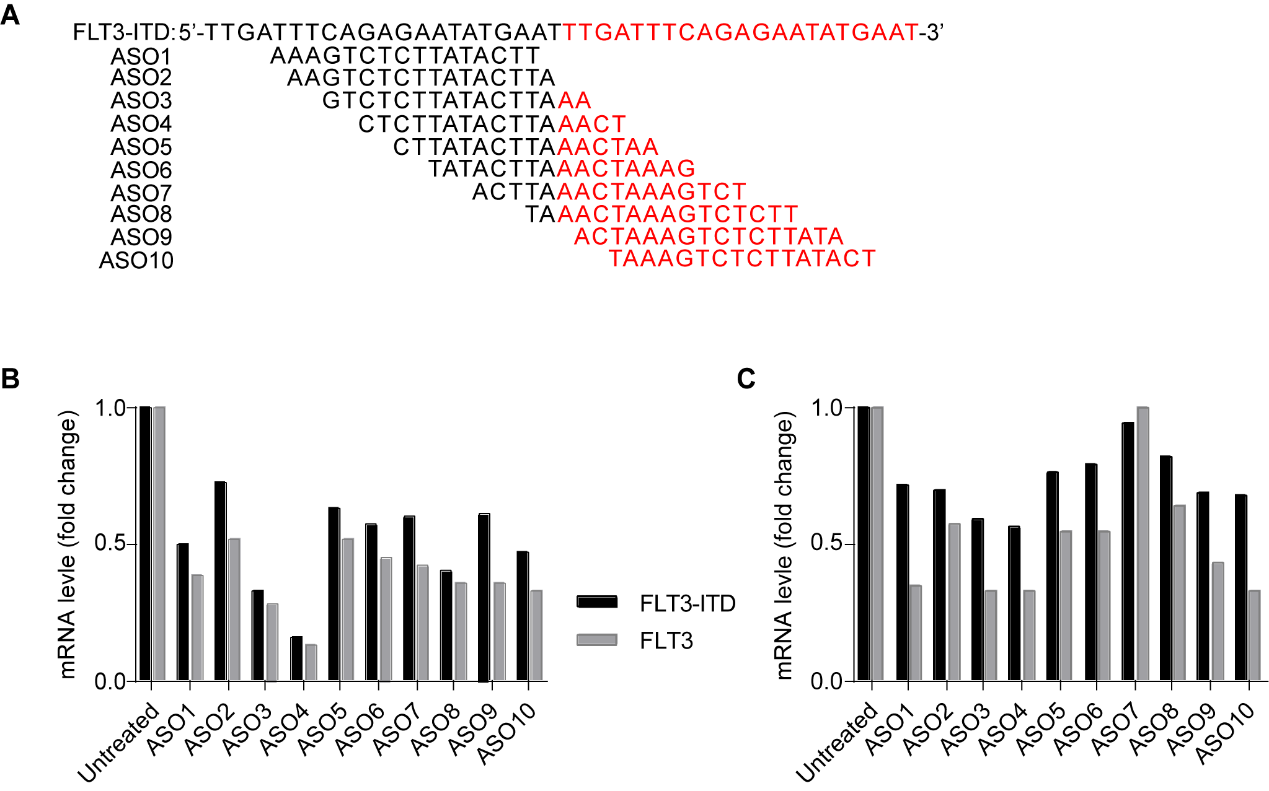


**Figure S3| Design and screening of FLT3-ITD ASOs.** (A) Detail sequences of 10 ASOs targeted FLT3-ITD mutation of MOLM13 cells. Black color sequences came from wild *FLT3* and Red color sequences came from the ITD mutation. (B) Test and screening of FLT3-ITD ASOs in MOLM13 cells at 24 hrs. qPCR analysis of *FLT3-ITD* and *FLT3* expression in MOLM13 cells treated or untreated with FLT3-ITD ASO loaded RBCEVs, relative to *GAPDH* internal control. (C)Test and screening of FLT3-ITD ASOs in MOLM13 cells at 48 hrs following above protocol.
